# O-linked N-acetylglucosamine transferase enhances secretory clusterin expression via liver X receptors and sterol response element binding protein regulation in cervical cancer

**DOI:** 10.18632/oncotarget.23588

**Published:** 2017-12-21

**Authors:** Min Jun Kim, Mee Young Choi, Dong Hoon Lee, Gu Seob Roh, Hyun Joon Kim, Sang Soo Kang, Gyeong Jae Cho, Yoon Sook Kim, Wan Sung Choi

**Affiliations:** ^1^ Department of Anatomy and Convergence Medical Science, Institute of Health Sciences, College of Medicine, Gyeongsang National University, Jinju, Gyeongnam, Republic of Korea

**Keywords:** O-GlcNAcylation, OGT, LXRs, SREBP-1, cisplatin

## Abstract

O-linked N-acetylglucosamine transferase (OGT) expression is increased in various cancer types, indicating the potential importance of O-GlcNAcylation in tumorigenesis. Secretory clusterin (sCLU) is involved in cancer cell proliferation and drug resistance, and recently, liver X receptors (LXRs) and sterol response element binding protein-1 (SREBP-1) were reported to regulate sCLU transcription. Here, we found that sCLU is significantly increased in cervical cancer cell lines, which have higher expression levels of O-GlcNAc and OGT than keratinocytes. OGT knockdown decreased expression of LXRs, SREBP-1 and sCLU through hypo-O-GlcNAcylation of LXRs. Additionally, treatment with Thiamet G, O-GlcNAcase OGA inhibitor, increased expression of O-GlcNAcylation and sCLU, and high glucose increased levels of LXRs, SREBP-1 and sCLU in HeLa cells. Moreover, OGT knockdown induced G_0_/G_1_ phase cell cycle arrest and late apoptosis in cisplatin-treated HeLa cells, and decreased viability compared to OGT intact HeLa cells. Taken together, these findings suggest that OGT, O-GlcNAcylated LXRs, and SREBP-1 increase sCLU expression in cervical cancer cells, which contributes to drug resistance.

## INTRODUCTION

Clusterin (CLU) is a highly conserved glycoprotein expressed ubiquitously in various tissue types, and has been implicated in aging, cancer progression, and several metabolic diseases [1, 2]. The isoforms of CLU, secretory CLU (sCLU) and nuclear CLU (nCLU), have different roles in disease, including certain cancers [3, 4]. sCLU acts as a molecular chaperone and promotes cell survival [5, 6], while sCLU is partially responsible for increased resistance of cancer cells to several chemotherapies due to its pro-survival functions [7–10]. In particular, CLU expression is regulated by metabolic signals, such as sterol regulatory element binding protein-1 (SREBP-1) [11–13], and hyperglycemia induces sCLU expression through the SREBP response element (SRE) but not by carbohydrate responsive element binding protein (ChREBP) in primary hepatocytes and hepatoma cell lines [14]. However, upstream regulation of SREBP-1, which induces sCLU expression has not yet been elucidated.

O-GlcNAcylation, a posttranslational modification, is thought to modulate a wide range of biological processes, such as transcription, cell growth, signal transduction, and cell motility [15–17]. O-GlcNAcylation is catalysed by the nucleocytoplasmic enzymes, O-linked-N-acetylglucosamine transferase (OGT) and O-GlcNAcase (OGA), which add or remove O-GlcNAc moieties, respectively [18]. Abnormal regulation of O-GlcNAcylation is implicated in multiple diseases, such as diabetes, Alzheimer's disease, and cancer [19–21]. Recent studies indicate that increased O-GlcNAcylation is general feature of cancer and contributes to invasion and metastasis in breast, prostate, lung, colorectal, and liver cancers [22–25]. The role of O-GlcNAcylation in tumorigenesis and progression is becoming clearer, but several unanswered questions, such as its role in drug resistance, still remain.

Recently, it was reported that high glucose levels stimulate and accelerate tumorigenesis in hepatocellular carcinoma cells through O-GlcNAcylation of liver X receptor (LXR) α and β [26]. LXRs are posttranslationally modified by O-GlcNAc in response to glucose, which in turn potentiates their transactivation of SREBP-1c [27]. Hyper-O-GlcNAcylation during hyperglycaemia induces a metabolic shift in highly proliferative cancer cells to lipogenesis and other tumorigenic processes. However, the role of O-GlcNAcylation in the transcriptional regulation of sCLU has not yet been investigated.

In this study, the relationship between O-GlcNA cylation and drug resistance in cervical cancer cells was examined. The results demonstrate that upregulation of LXR O-GlcNAcylation enhances sCLU expression through increased expression of SREBP-1, which induces drug resistance in cervical cancer cells.

## RESULTS

### Expression levels of O-GlcNAc, OGT, and sCLU are elevated in cervical cancer cell lines and cervical cancer tissues

**Figure 1:**
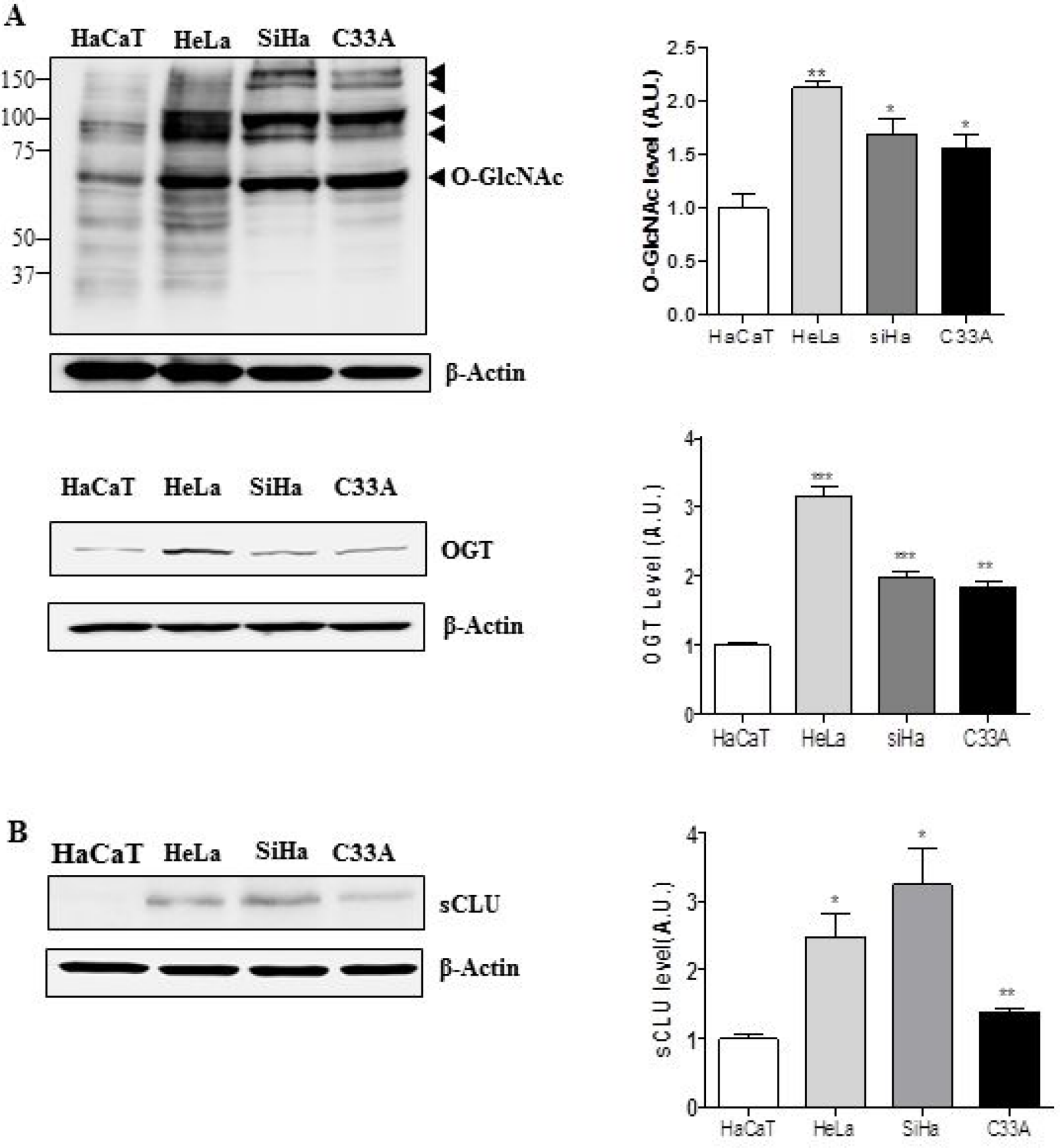
Expression of sCLU, O-GlcNAc, and OGT is elevated in cervical cancer cells Expression of O-GlcNAc, OGT (**A**), and sCLU (**B**) was analysed by western blot in cervical cancer cell lines (HeLa, SiHa, and C33A) and normal keratinocytes (HaCaT). Black arrowheads indicate the specific signals for each protein. Blots were reprobed for β-actin to ensure equivalent loading; quantification of each band was normalized to the β-actin signal. Data are presented as mean ± S.E.M. (*n* = 3). ^*^p < 0.05 and ^**^p < 0.01 relative to levels in HaCaT cells.

**Figure 2:**
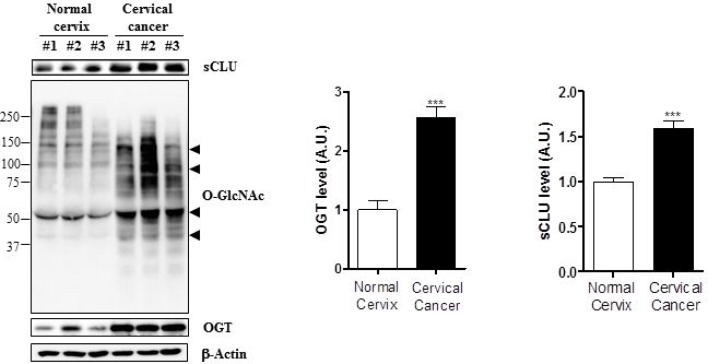
Expression of sCLU, O-GlcNAc, and OGT is increased in cervical cancer tissue Expression of O-GlcNAc, OGT, and sCLU was analysed by western blot in cervical cancer (*n* = 14) and normal cervical (*n* = 6) patient tissue. Black arrowheads indicate the specific signals for each protein. Blots were reprobed for β-actin to ensure equivalent loading and quantification of each band was normalized to the β-actin signal. Data are presented as mean ± S.E.M. (*n* = 3). ^***^p < 0.001 for normal cervix versus cervical cancer.

OGT and O-GlcNAcylation levels are elevated in several cancer types [21]. Therefore, we examined expression of O-GlcNAcylation and OGT in HeLa, Caski, and C33A cervical cancer cell lines. Western blot analysis revealed that O-GlcNAc and OGT levels were elevated in these three cervical cancer cell lines relative to a keratinocyte cell line, HaCaT (Figure 1A). Next, we examined whether cervical cancer cells express more sCLU than keratinocytes, and found that indeed, all three lines exhibited higher expression of sCLU than normal keratinocytes (Figure 1B). Quantification of O-GlcNAc, OGT, and sCLU in normal keratinocytes and cervical cancer cell lines is shown in Figure 1B. To show whether mRNA levels of those genes are changed in cervical cancer cells, we performed polymerase chain reaction (PCR) experiment. Indeed, we found that levels of sCLU and OGT mRNA expression were increased in HeLa, SiHa, C33A compared to HaCaT cells (Supplementary Figure 1). Additionally, we examined expression of O-GlcNAc, OGT and sCLU in normal and cancer tissues. We found that sCLU expression was increased in cervical cancer tissue, which had higher O-GlcNAc and OGT expression than normal cervical tissue (Figure 2). Together, these data suggest that OGT, O-GlcNAc, and sCLU expression is increased in cervical cancer cell lines and cervical cancer.

### O-GlcNAcylation increases sCLU expression in HeLa cells

**Figure 3:**
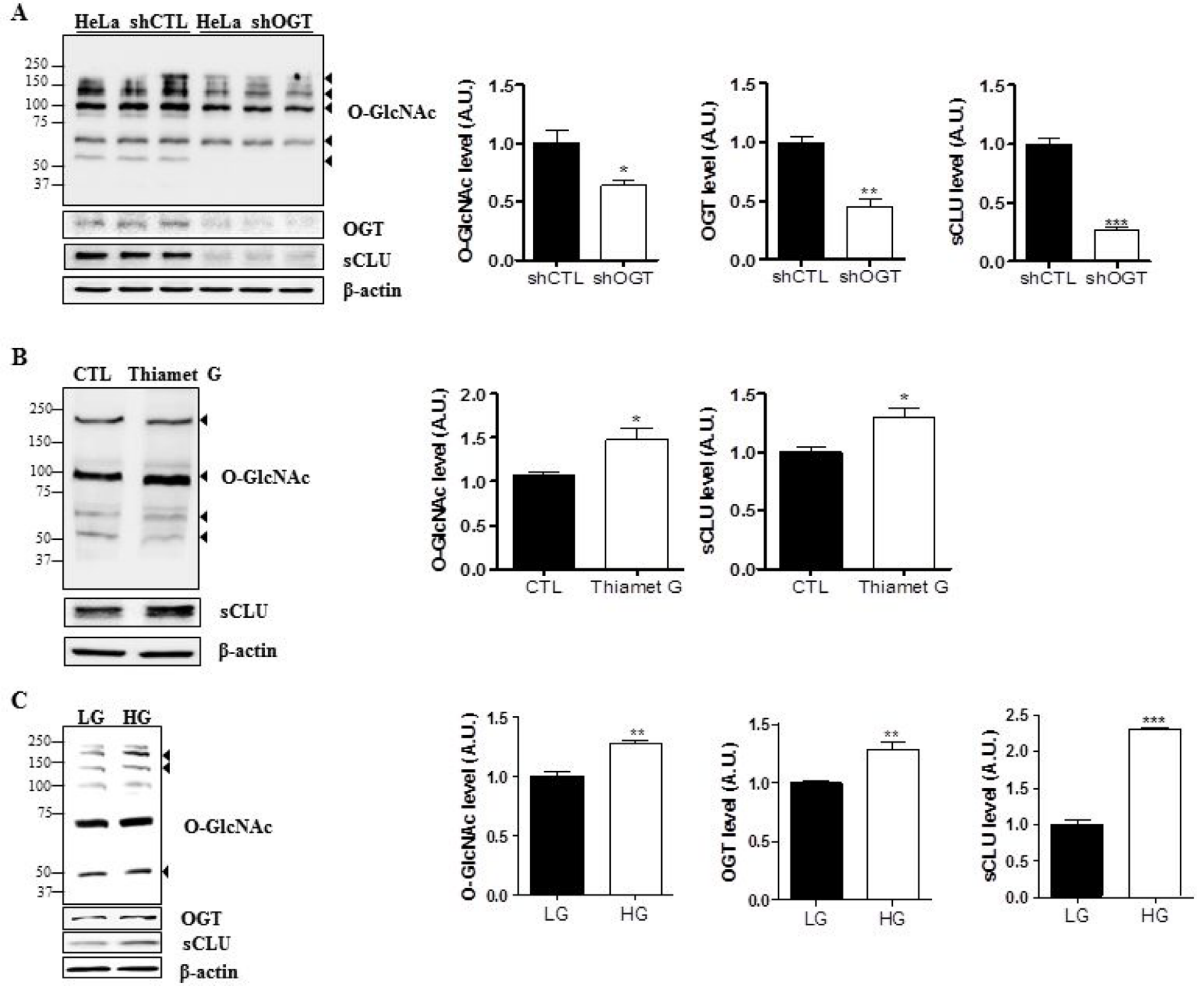
O-GlcNAcylation regulates sCLU expression in HeLa cells (**A**) Expression of sCLU was measured by western blot in lentivirus-mediated shOGT or shCTL HeLa cells. (**B**) HeLa cells were treated with Thiamet G (OGA inhibitor; 100 μM) for 24 h, and sCLU expression level was determined by western blot analysis. (**C**) HeLa cells were incubated for 24 h under low-glucose (LG; 5.5 mM) or high-glucose, (HG; 25 mM) conditions, and total proteins were isolated and analysed by western blot. For all panels, band intensity was normalized to the β-actin loading control. Data are presented as mean ± S.E.M. (*n* = 3). ^*^p < 0.05, ^**^p < 0.01, ^***^ and *p* < 0.001, relative to the levels of expression in the indicated control condition.

To examine the effects of O-GlcNAcylation on sCLU expression, we engineered control and OGT-knockdown HeLa cells. Cell lines were constructed by transfection of a scrambled shRNA (shCTL) or OGT-specific shRNA (shOGT) using a lentiviral delivery system. First, we confirmed downregulation of O-GlcNAc and OGT after shOGT transfection by western blot analysis. Next, we examined sCLU levels, and found that the sCLU was significantly decreased in cells transfected with shOGT compared with shCTL cells (Figure 3A). The quantification of O-GlcNAc, OGT, and sCLU in control and OGT-deficient HeLa cells is shown in Figure 3A.

To test whether OGT regulates sCLU, HeLa cells were treated with a highly selective OGA inhibitor, Thiamet G, to stimulate O-GlcNAcylation. Treatment with Thiamet G markedly elevated levels of O-GlcNAcylation and sCLU expression in HeLa cells (Figure 3B).

Because it is generally accepted that high glucose induces hyper-O-GlcNAcylation *in vitro* [28], we next grew HeLa cells in serum-free medium containing 5.5 mM glucose (LG) or 25 mM glucose (HG) for 24 h, and found that O-GlcNAcylation and OGT expression were enhanced in HeLa cells exposed to HG. In addition, we examined expressions of OGT and O-GlcNAc under no glucose without serum. We found that expressions of OGT and O-GlcNAc were decreased by glucose depletion (Supplementary Figure 2). Next, we examined sCLU expression in HeLa cells exposed to HG by immunoblot, and found that expression of sCLU increased more than two-fold in HeLa cells exposed to HG compared with those exposed to LG (Figure 3C). These results indicate that high glucose-induced O-GlcNAcylation upregulation enhances sCLU expression in HeLa cells.

### O-GlcNAcylation increases expression of LXRs and SREBP-1

**Figure 4:**
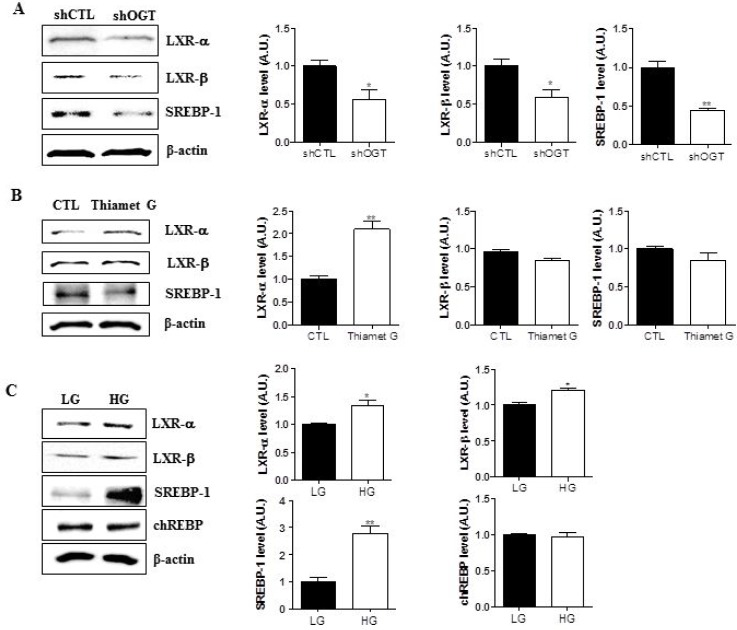
O-GlcNAc regulates expression LXR-α, LXR-β, and SREBP-1 in HeLa cells Three experimental conditions (OGT knockdown (**A**)) and Thiamet G treatments (**B**), and hyperglycemia (**C**)) were used to examine regulation of LXR-α, LXR-β, and SREBP-1 expression. Western blot analysis was conducted using LXR-α, LXR-β, and SREBP-1 antibodies and β-actin was used as a loading control. For densitometry analysis, band intensity was normalized to the β-actin signal. Data are presented as mean ± S.E.M. (*n* = 3). ^*^p < 0.05 and ^**^p < 0.01.

To test whether targeting OGT affects expression of LXR-α, LXR-β, and SREBP-1, we measured their expression in HeLa cells transfected with control (shCTL) or OGT-specific shRNA (shOGT) constructs by immunoblot. We found that OGT knockdown in HeLa cells decreased the expression of LXR-α, LXR-β, and SREBP-1 (Figure 4A, ^*^p < 0.05, ^**^p < 0.01). Moreover, when HeLa cells were treated with Thiamet G, levels of LXR-α were increased (Figure 4B, ^**^p < 0.01). To evaluate the effects of glucose on the system, HeLa cells were grown in serum-free medium containing 5.5 mM glucose (LG) or 25 mM glucose (HG) for 24 h. Expression of LXR-α, LXR-β, and SREBP-1 was significantly higher in HG conditions than in LG conditions (Figure 4C, ^*^p < 0.05, ^**^p < 0.01). However, ChREBP expression levels remained unaltered in HeLa cells exposed to hyperglycaemic conditions. Therefore, these data suggest that O-GlcNAcylation increases expression of LXRs and SREBP-1.

### Targeting OGT decreases O-GlcNAcylated LXR-α and LXR-β

**Figure 5:**
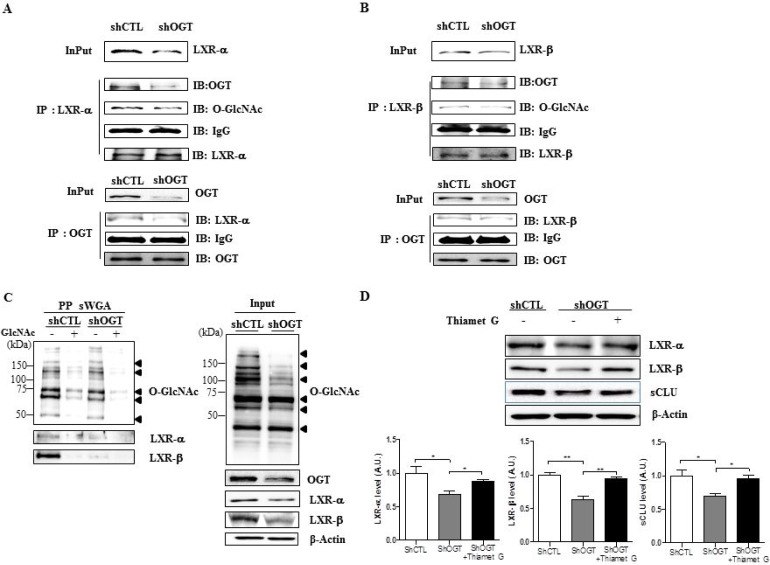
OGT knockdown decreases O-GlcNAcylation of LXR-α and LXR-β Cellular extracts were subjected to immunoprecipitation with LXR-α (**A**) or LXR-β (**B**) antibodies, followed by immunoblot with anti-O-GlcNAc or OGT antibodies. (**C**) Cellular extracts were subjected to immunoprecipitation with anti-O-GlcNAc or -OGT antibodies, followed by western blot analysis with anti-LXR-α or -LXR-β antibodies. β-actin served as a loading control. (**D**) Levels of LXR-α, LXR-β and sCLU in OGT knockdown HeLa cells treated with Thiamet G. Data are presented as mean ± S.E.M. (*n* = 3). ^*^p < 0.05 and ^**^p < 0.01.

The effects of OGT targeting on O-GlcNAcylation of LXR-α and -β were analysed by immunoprecipitation and succinylated wheat germ agglutinin affinity (sWGA) immunoprecipitation assays. Cell extracts were subjected to immunoprecipitation with LXR-α or LXR-β antibodies, followed by immunoblot analysis with anti-O-GlcNAc or OGT antibodies. We observed increased interaction of OGT with LXR-α or LXR-β, as well as O-GlcNAcylation of LXRs, in shCTL HeLa cells, but not in shOGT HeLa cells (Figure 5A and 5B).

We confirmed the composition of the GlcNAc with a sWGA purification assay in HeLa cells transfected with shCTL or shOGT. Upon addition of an inhibitory monosaccharide N-GlcNAc during the sWGA-lectin-affinity purification step, O-GlcNAc nearly disappeared (Figure 5C). Our data show that O-GlcNAcylated LXR-α and LXR-β were significantly decreased in OGT-knockdown HeLa cells relative to the control. Then, to confirm whether O-GlcNAcylation regulates LXRs expression, we treated OGT knockdown HeLa cells with Thiamet G. As expected, we found that levels of LXRs and sCLU were significantly increased in OGT knockdown HeLa cells treated with Thiamet G compared to control cells (Figure 5D). Taken together, our results suggest that upregulated O-GlcNAcylated LXRs may induce sCLU expression.

### OGT knockdown increases cell cycle arrest and apoptosis of HeLa cells upon cisplatin treatment

**Figure 6:**
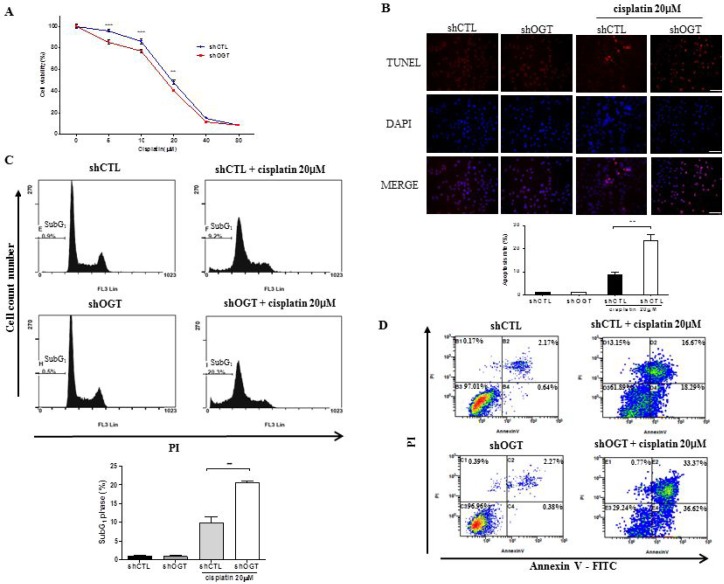
OGT knockdown increases sensitivity of HeLa cells to cisplatin (**A**) HeLa cells transfected with non-specific (shCTL) or OGT-specific shRNA (shOGT) constructs were treated with different concentrations of cisplatin (5, 10, 20, 40, or 80 μM) for 24 h. Cell metabolic activity was measured using a MTT assay to determine the rate of HeLa cell death in response to cisplatin. Percent cell survival is presented as mean ± S.E.M. (*n* = 16). ^**^p < 0.01 and ^***^p < 0.001, vs. shCTL. (**B**) HeLa cells transfected with shCTL or shOGT were treated with 20 μM of cisplatin for 24 h and stained with TUNEL. Percentages of TUNEL-positive cells are presented as mean ± S.E.M. (*n* = 3). ^**^p < 0.01. (**C**) shCTL HeLa and shOGT HeLa cells (1 × 10^5^ cells/ml) were treated with cisplatin (20 μM) for 24 h. Following treatment, cells were harvested, fixed, and stained with PI, then analysed by flow cytometry. Bar diagram indicates the percentage of cells in the SubG_1_ phase of the cell cycle. Data are presented as mean ± S.E.M. (*n* = 3). ^**^p < 0.01. (**D**) shCTL HeLa or shOGT HeLa cells were stained with Annexin V-FITC and PI after cisplatin treatment, then analysed by flow cytometry. Experiments were performed three times separately.

To determine the effect of OGT on sensitivity to cisplatin, we carried out a MTT assay, TUNEL stain, and fluorescence-activated cell sorting (FACS) analysis to measure cell metabolism and viability in HeLa cells transfected with shCTL or shOGT after cisplatin treatment. shOGT HeLa cells showed enhanced cisplatin sensitivity compared to shCTL HeLa cells (Figure 6A, ^***^p < 0.001). To examine the effects of OGT knockdown on cisplatin-induced apoptosis, shCTL and shOGT HeLa cells were treated with 20 μM cisplatin for 24 h and apoptosis was analysed using TUNEL staining and FACS analysis (Figure 6B, ^**^p < 0.01 and Figure 6C, ^**^p < 0.01). We found that depletion of OGT with an OGT-specific shRNA resulted in more than a two-fold increase in cisplatin-induced HeLa cell death. In particular, the combination treatment of an OGT-specific shRNA with cisplatin resulted in late apoptosis, as shown by FACS (Figure 6D).

To confirm whether increased O-GlcNAcylation rescues cell death, we treated HeLa cells with PUGNAc, OGA inhibitor, and then with cisplatin. FACS Analysis showed that cell death was rescued by PUGNAc (Supplementary Figure 3).

## DISCUSSION

This study demonstrates that levels of O-GlcNAcylation and OGT are elevated in cervical cancer cell lines. Secretory CLU (sCLU) is also increased in cervical cancer cells, together with LXRs and SREBP-1. O-GlcNAcylation, a posttranslational modification, affects several biological processes, including translation, the stress response, and nutrient sensing [29–31]. In cancer, hyper-O-GlcNAcylation occurs in prostate, liver, breast, lung, and colon cancers [22–25]. Here, we confirmed that OGT overexpression and hyper-O-GlcNAcylation occur in cervical cancer cells.

CLU can act as both an apoptotic or anti-apoptotic factor, and these opposing functions are mediated by two different isoforms, sCLU and nCLU [3, 4]. sCLU is a pro-survival factor that is overexpressed in various human cancers, including prostate, breast, lung, ovarian, and cervical cancer [9, 32–34]. In particular, sCLU expression in cervical cell cells is associated with tumour progression, from primary to metastatic cancer, and correlates with cancer drug resistance [35–37]. However, the regulatory mechanism controlling sCLU in cervical cancer remains unclear. In this study, we found that OGT and sCLU expression were elevated in cervical cancer cell lines, and that sCLU expression is regulated by O-GlcNAcylation. These findings suggest that inhibition of O-GlcNAcylation may be a viable strategy to target sCLU expression in cervical cancer.

sCLU expression is induced by insulin signalling through SREBP-1c and SREBP-1c-mediated activation of the CLU promoter upon recruitment of the transcription factor to a SRE in the CLU promoter [14]. In this study, high glucose concentrations induced CLU expression through increased SREBP-1c binding to SRE [14]. However, activation of ChREBP did not induce CLU. The authors concluded therefore that SREBP-1c is the key transcription factor in high glucose-induced CLU expression. In our study, we found that ChREBP expression was not involved in regulating sCLU expression in hyperglycaemic conditions (Figure 4), consistent with previous studies [14].

Until now, direct regulation of SREBP-1c by O-GlcNAcylation has not been reported. Here, we examined whether OGT regulates the expression of transcription factors upstream of SREBP-1c, like LXR-α and LXR-β [38–40]. LXR-α and LXR-β are regulated by O-GlcNAcylation and induce CLU expression [26, 27]; therefore, we hypothesized that OGT might induce SREBP-1 expression by upregulating O-GlcNAcylated LXRs. Indeed, we found that depletion of OGT decreased LXR stabilization, resulting in diminished SREBP-1 expression. Therefore, CLU expression can be regulated by enhanced SREBP-1 expression through O-GlcNAcylated LXRs.

Previous studies have shown that overexpression of sCLU in cervical cancer cells causes resistance to chemotherapeutic agents such as cisplatin, doxorubicin, and camptothecin [7–9]. In addition, sCLU promotes metastasis by enhancing cell motility, and sCLU silencing inhibits tumour growth and motility [35, 41]. Additionally, OGT depletion suppresses tumour growth in nude mice [42]. Currently, methods for silencing sCLU expression using antisense oligonucleotides have been developed and approved for clinical trials [35, 41]. Therefore, downregulation of sCLU is a primary goal for treating drug-resistant cancers.

**Figure 7:**
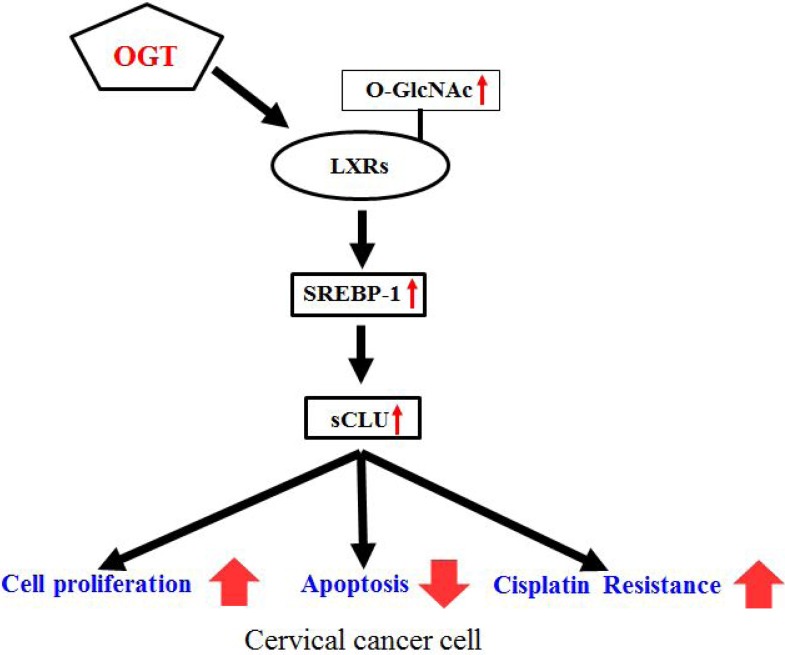
Schematic depicting the mechanism of OGT-induced cisplatin resistance by enhanced sCLU expression through O-GlcNAcylated LXRs Increased OGT enhances expression of O-GlcNAcylated LXRs, which increase sCLU expression through SREBP-1. Consequently, overexpressed sCLU enhances cisplatin resistance, cell proliferation, and decreases apoptosis.

Our results demonstrate that OGT knockdown completely blocks sCLU expression through O-GlcNAcylated LXRs. The pathway regulating sCLU expression by OGT is summarised in Figure 7. Together, our results indicate that the role of OGT in drug resistance is through regulation of sCLU through SREBP-1, thereby increasing resistance to cisplatin. OGT may be a target for cancer therapy in cancers where sCLU is upregulated. However, there is no drug available that completely blocks sCLU expression. This newly discovered mechanism could therefore be one of the pathways used for drug development to overcome chemotherapy resistance. We propose that OGT inhibitors or antagonists may be the most promising future therapeutics.

## MATERIALS AND METHODS

### Materials

Dulbecco's modified Eagle medium (DMEM), penicillin/streptomycin, and fetal bovine serum were obtained from Gibco (Invitrogen, Carlsbad, CA, USA). Dimethyl sulfoxide, methylthiazoltetrazolium (MTT) and cisplatin were purchased from Sigma (Saint Louis, MO, USA). Antibodies against OGT, SREBP-1 and clusterin-α were from Santa Cruz Biotechnology (CA, USA). ChREBP antibodies were from NOVUS (Cambridge, UK). β-actin antibodies were from PIERCE (Rockford, IL, USA), O-GlcNAc antibodies were from Thermo Fisher Scientific Inc. (Rockford, IL, USA), and anti-LXR-α and anti-LXR-β antibodies were from R&D Systems.

### Lentiviral shRNA production and infection

Lentiviruses expressing shRNA against OGT (shOGT) or scrambled shRNA (shCTL) were made as follows. Lentiviral pLKO.1-puro vectors were purchased from Sigma. The human OGT shRNA sequence (TRCN0000035064) was: 5′-CCGG-GCCCTAAGTTTGA GTCCAAAT-CTCGAG-ATTTGGACTCAAACTTAGG GC-TTTTTG-3′. The scrambled shRNA sequence (Product No. SHC002V) was: 5′-CCGG-CAACAAGATGAAGAG CACCAA-CTCGAG-TTGGTGCTCTTCATCTTGTTG TTTTT-3′. For lentivirus production, HEK 293T cells were transfected with the pLKO.1 vector, along with packaging plasmids encoding Gag/Pol, Rev, and VSV-G, using Lipofectamine 2000 (Invitrogen, Carlsbad, CA, USA) according to the manufacturer's instructions. Culture media containing lentiviral particles was collected and filtered 48 h or 72 h after transfection. Virus-containing supernatants were pooled and stored at −80°C. HeLa cells were infected with viruses in medium and selected for stable expression of shRNA with puromycin (10 μg/mL) treatment for two weeks.

### Cell lines

Cervical cancer cell lines [HeLa (HPV-18-positive), SiHa (HPV-16-positive), and C33A (HPV-negative)] and a human keratinocyte cell line (HaCaT) were obtained from American Type Culture Collection (Manassas, VA, USA). HeLa and HaCaT cells were maintained in DMEM. SiHa and C33A cells were maintained in Minimum Essential Medium. All cells were cultured in 5% CO_2_ at 37°C. All media were supplemented with 10% fetal bovine serum (Invitrogen, Carlsbad, CA, USA), 100 μg/mL streptomycin, and 100 units/mL penicillin (Invitrogen, Carlsbad, CA, USA). For glucose experiments, cells were washed twice with PBS and then incubated in low glucose media with out serum for 3 h. After incubation, cells were exposed to low (5.5 mM) or high (25 mM) glucose media without serum for 24 h.

### Patient tumour samples

Human cervical tissues were collected from patients undergoing cervical biopsies and loop electrosurgical excision procedures. The patients were high-risk HPV16/18-positive and aged between 29 and 84 years (mean, 57.1 years). Tissue samples from normal (*n* = 6) or cervical cancer patients (*n* = 14) were used for protein extraction. Informed consent was obtained from all participants, and the study was approved by the Ethics Committee of our hospital (IRB No.2014-10-024-001).

### Western blot analysis

The proteins from cell lysates were separated by SDS-PAGE on 8–12% gels and transferred to nitrocellulose membranes. All gels were run under the same voltage conditions. Following transfer, the membrane was incubated with primary antibodies, including O-GlcNAc (RL2) (1:5000), OGT (1:1000), clusterin-α (1:1000), SREBP-1 (1:5000), LXR-α (1:1000), LXR-β (1:1000), ChREBP (1:1000), PARP (1:1000), cleaved PARP (1:1000) and β-actin (1:10000) overnight at 4°C. Chemiluminescence (ECL, Amersham Biosciences, Piscataway, NJ) was used to detect protein bands. Blot images were captures on a RAS-4000 image reader (Fujifilm, Japan).

### Immunoprecipitation

Protein extracts were mixed with protein A/G agarose beads (Santa Cruz Biotechnology, CA, USA), incubated for 1 h at 4°C, then centrifuged at 12,000 × *g* for 1 min. The supernatant was incubated with the immunoprecipitation antibodies overnight at 4°C and incubated with protein A/G agarose beads for 2 h at 4°C. The negative control was prepared with protein A/G agarose beads without antibody. The protein-bead complex was washed and collected by centrifugation. Samples were boiled in loading buffer to remove agarose beads, and proteins (2 mg) were resolved on 10% SDS-PAGE gels. Proteins were transferred to membranes and probed with antibodies against the interacting protein of interest as described above (see western blotting methods).

### Succinylated wheat germ agglutinin (sWGA) affinity purification

HeLa cells were lysed with RIPA lysis buffer (150 mM NaCl, 50 mM Tris, pH 7.4, 1 mM EDTA, and 0.5% Nonidet P-40), and cell lysates (200 μg of protein) were incubated with agarose-conjugated sWGA beads (Vector Laboratories, Burlingame, CA, USA) overnight at 4°C. For control samples, the inhibitory monosaccharide GlcNAc was added during the sWGA-lectin-affinity purification. Precipitates were washed three times with RIPA buffer and proteins were eluted by boiling in SDS sample buffer. Lysates were then analysed by western blot.

### Cell proliferation assay

HeLa shCTL or HeLa shOGT cells were seeded at a density of 1.0 × 10^4^ cells/well in a 96-well plate, and a MTT assay was conducted after cisplatin treatment for the indicated times (0 h, 24 h, and 48 h). The concentrations of cisplatin used were 0, 5, 10, 20, 40, and 80 μM. MTT solution (2 mg/mL) was added to each well, and the plates were incubated at 37°C for 2 h. The resulting formazan crystals were dissolved in dimethyl sulfoxide, and the absorbance of the solution was measured at 570 nm using a microplate reader (Tecan, Maennedorf, Switzerland).

### Immunocytochemistry and TUNEL staining

shCTL and shOGT HeLa cells were seeded into 24-well plates at 2 × 10^4^ cells per well, then grown for 16 h. Next, cells were treated with 20 μM cisplatin for 24 h, washed with phosphate-buffered saline (PBS), fixed with cold 4% paraformaldehyde, and permeabilised for 2 min with 0.5% Triton X-100 and 0.05% sodium azide in 0.05 M PBS on ice. Apoptotic cells were measured using an *in situ* cell death detection kit, stained with TMR red (Roche Applied Science, Mannheim, Germany) for 15 min, and mounted on slides using ProLong Gold Antifade reagent (Invitrogen, Carlsbad, CA, USA) for a nuclear stain. All images were taken using a fluorescence microscope (BX51- DSU; Olympus, Tokyo).

### Cell cycle and apoptosis analysis

Cells were collected and seeded into 6-well plates at 1 × 10^5^ cells per well and cultured for 16 h. Next, cells were treated with 20 μM cisplatin for 24 h, then trypsinised, fixed with cold 90% ethanol, and incubated for 1 h at 4°C. Cells were pelleted and resuspended in 1 ml PBS, containing propidium iodide (1 mg/ml) and RNase A (1 mg/ml). Following incubation at 37°C for 30 min, cell cycle and apoptosis (subG_1_) were determined by flow cytometry according to the manufacturer's protocol (FACscan, BD Bioscience). Analyses were performed using CXP 2.2 software. All experiments were performed three times separately.

### Detection of apoptotic cells by Annexin-V-FITC/propidium iodide (PI) double staining

Induction of apoptosis was examined by FACS analysis with Annexin-V-FITC and PI double staining. Cells were collected and seeded into 6-well plates at 1 × 10^5^ cells per well, then cultured for 16 h. Next, cells were treated with 20 μM cisplatin for 24 h, then trypsinised, fixed with cold 90% ethanol, and incubated for 1 h at 4°C. Cells were pelleted and resuspended in binding buffer for 15 min at room temperature in the dark. Apoptotic cells were detected by flow cytometry (FACscan, BD Bioscience). Analyses were performed using CXP 2.2 software. All experiments were performed three times separately.

### Statistical analysis

Data are expressed as the mean ± standard error of the mean (S.E.M). Statistical significance was determined using the Student's *t*-test to compare two groups and ANOVA to compare multiple treatment groups (GraphPad Prism, La Jolla, CA, USA). *p* values < 0.05 were considered statistically significant.

## SUPPLEMENTARY MATERIALS FIGURES


