# Supplementary material for: O-linked N-acetylglucosamine transferase enhances secretory clusterin expression via liver X receptors and sterol response element binding protein regulation in cervical cancer
Source: Oncotarget. 2017 Dec 21;9(4):4625–36. doi: 10.18632/oncotarget.23588 (PMC5797001; doi:10.18632/oncotarget.23588)
Supplement: Supplementary file 1 [file oncotarget-09-4625-s001.pdf]

O-linked N-acetylglucosamine transferase enhances secretory clusterin expression via liver X receptors and sterol response element binding protein regulation in cervical cancer

SUPPLEMENTARY MATERIALS

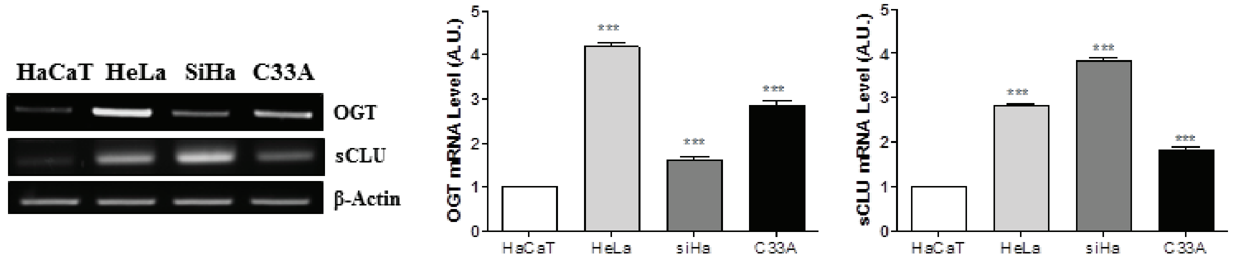

Supplementary Figure 1: Expression levels of sCLU and OGT mRNA expression are increased in cervical cancer cell lines (HeLa,SiHa,C33A) compared to HaCaT cells. mRNA expression levels of sCLU and OGT are analysed by RT-PCR respectively (n = 3). Data are presented as mean ± S.E.M. (n = 3). $^{***}p < 0.001$ The oligonucleotide primers used for RT-PCR were as follows: OGT, 5′-CGGGCTATCGAACTACAACCA-3′ (forward) and 5′- CCCATATTAGAGTAGGCATCAGCAAAG-3′ (reverse); sCLU, 5′-AGATCAGCGCCTGAGAAGCT-3′ (forward) and 5′- GGGACCAGTGTACCTTCTCG-3′ (reverse) ; β-actin, 5′-CTGGCACC ACACCTTCTACAATG-3′ (forward) and 5′- CCTCGTAGATGGGCACAGTGTG-3′ (reverse). OGT cycle- 25cycle 35cycle, β-actin-20 cycle.

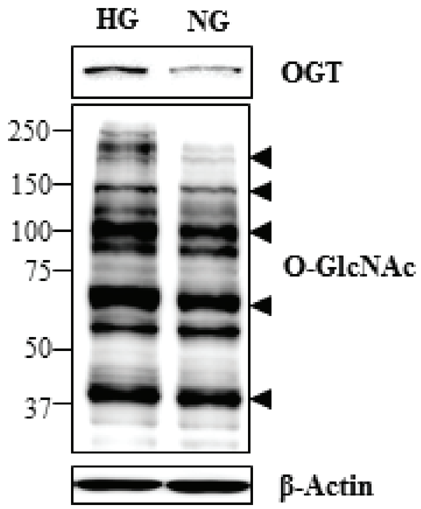

Supplementary Figure 2: Expressions of OGT and O-G1cNAc are decreased by glucose depletion condition. HeLa cells were incubated for 24 h under no glucose (NG) with serum free or high-glucose (HG; 25 mM) with serum free conditions. Total proteins were extracted and analysed by western blot, respectively (n = 3).

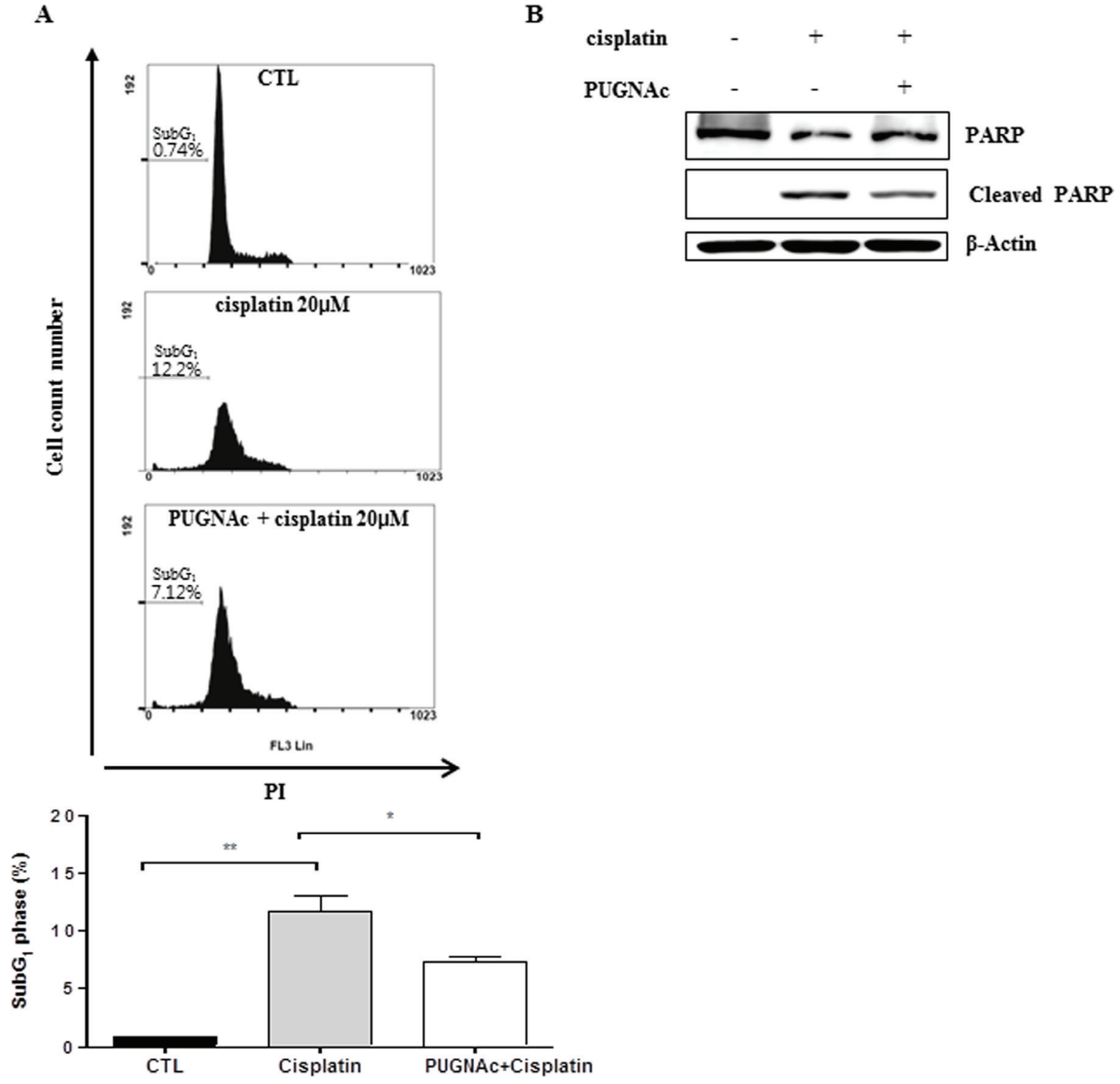

Supplementary Figure 3: Increased O-GlcNAcylation rescues cell death in HeLa cell. (**A**) HeLa cells (1×10^5 cells/ml) treated with OGA inhibitor, PUGNAc (100 μM) for 24 h or after following treatment HeLa cells were treated with cisplatin (20 μM) for 24 h again. After that cells were harvested, fixed and stained with PI, then analysed by flow cytometry. Bar diagram indicates the percentage of cells in the $SubG_1$ phase of the cell cycle. Data are presented as mean ± S.E.M. ($n = 3$). $^{*}p < 0.05$, $^{**}p < 0.01$ (**B**) Expression of PARP, cleaved PARP was analysed by western blot in HeLa cells treated with cisplatin for 24 h, after PUGNAc treatment.
